# The Influence of Supplementation of Anthocyanins on Obesity-Associated Comorbidities: A Concise Review

**DOI:** 10.3390/foods9060687

**Published:** 2020-05-26

**Authors:** Bhagavathi Sundaram Sivamaruthi, Periyanaina Kesika, Chaiyavat Chaiyasut

**Affiliations:** Innovation Center for Holistic Health, Nutraceuticals, and Cosmeceuticals, Faculty of Pharmacy, Chiang Mai University, Chiang Mai 50200, Thailand; p.kesika@gmail.com

**Keywords:** anthocyanins, obesity, cyanidin-3-*O*-β-D-glucoside, body weight, chronic diseases

## Abstract

Anthocyanins are water-soluble plant pigments, and based on their chemical structure (nature, position, and the number of sugar moieties attached; the number of hydroxyl groups; acylation of sugars with acids) about 635 different anthocyanins have been identified and reported from plants. Cyanidin, peonidin, pelargonidin, petunidin, and malvidin are the commonly found anthocyanidins (aglycon forms of anthocyanins) in edible plants out of almost 25 anthocyanidins that are identified (based on the position of methoxyl and hydroxyl groups in the rings) in nature. Anthocyanins are known for numerous health benefits including anti-diabetes, anti-obesity, anti-inflammatory bowel disease, anti-cancer, etc. Obesity can be defined as excessive or abnormal adipose tissue and body mass, which increases the risk of developing chronic diseases such as diabetes, cardiovascular diseases, cancers, etc. The manuscript summarizes the recent updates in the effects of anthocyanins supplementation on the health status of obese subjects, and briefly the results of in vitro and in vivo studies. Several studies confirmed that the consumption of anthocyanins-rich food improved obesity-associated dysbiosis in gut microbiota and inflammation in adipose tissue. Anthocyanin consumption prevents obesity in healthy subjects, and aids in maintaining or reducing the body weight of obese subjects, also improving the metabolism and energy balance. Though preclinical studies proved the beneficial effects of anthocyanins such as the fact that daily intake of anthocyanin rich fruits and vegetables might aid weight maintenance in every healthy individual, Juҫara pulp might control the inflammatory status of obesity, Queen garnet plum juice reduced the blood pressure and risk factors associated with metabolic disorders, and highbush organic blueberries improved the metabolism of obese individuals, we don’t have an established treatment procedure to prevent or manage the over-weight condition and its comorbidities. Thus, further studies on the optimum dose, duration, and mode of supplementation of anthocyanins are required to develop an anthocyanins-based clinical procedure.

## 1. Introduction

Obesity is one of the known health impairments that reduces the quality of life. The prevalence of obesity is increasing drastically, and the incidence of obesity has tripled since 1975. As of 2016, about 650 million people globally were found to be obese [1,2]. Energy imbalance is the fundamental cause of obesity. Generally, consumption of high energy foods, lack of physical activities, lifestyle changes, gut microbiota, and increased urbanization are the reasons for obesity. The oxidative stress and chronic low-grade inflammation in adipose tissue are also associated with obese-related comorbidities [3]. Obesity is associated with the development of several diseases including diabetes, cardiovascular diseases, inflammatory bowel disease, non-alcoholic fatty liver disease, pancreatitis, Alzheimer’s and Parkinson’s diseases, cancers, etc. [4,5,6,7,8,9,10].

Dietary therapy, planned regular physical activity, and surgical procedures are effective measures for the treatment of obesity. Weight loss of about 2–3, 4–6, and 20–40 kg can be achieved within one to two years duration by exercise therapy, dietary therapy, and bariatric surgery, respectively [11]. The studies revealed that the supplementation of probiotics and, more generally, phenolic compounds such as flavonoids and anthocyanins improved the health status of obese people [3,12,13,14].

**Figure 1:**
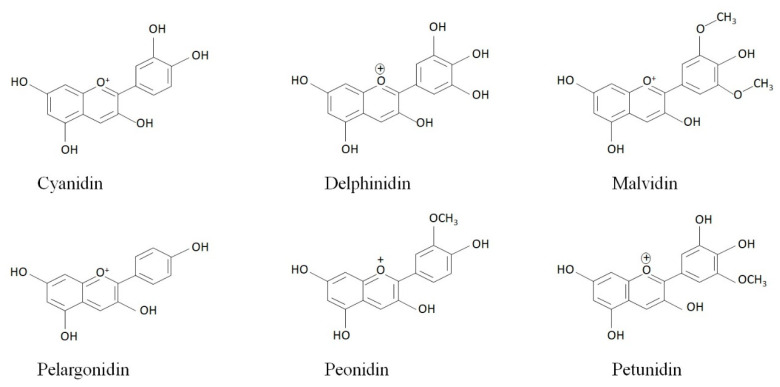
Common anthocyanidins in nature.

Anthocyanins are water-soluble plant pigments responsible for the red, blue and purple color of many plant parts. Anthocyanins are generally richly found in flowers and fruit bodies of several plants including berries, plums, grapes, and vegetables like purple cabbage and red potatoes [15]. Anthocyanins are found in glycosides and acylated forms. Glucosides are the most common glycoside forms that are present in almost all anthocyanin-rich plant parts. Diglucosides are the common glycoside forms found in red fruits such as jujube (Cyanidin-3,5-diglucoside, delphinidin-3,5-diglucoside, and pelargonidin-3,5-diglucoside) [16], pomegranate (Cyanidin-3,5-diglucoside, delphinidin-3,5-diglucoside) [17,18], and red-jumbo (Cyanidin-3,5-diglucoside,) [19]. Acylated forms of anthocyanins are further classified as coumaroylated, caffeoylated and malonylated anthocyanin. The aglycone form of anthocyanins are called anthocyanidins. Cyanidin (50%), delphinidin (12%), peonidin (12%), malvidin (7%), petunidin (12%), and pelargonidin (7%) are the most common anthocyanidins [20] (Figure 1).

Anthocyanins are present in most fruits (apple, apricot, bilberry, blackberry, blackcurrant, blueberry, cherry, cranberry, grape, haskap berry, mulberry, orange, peach, plum, pomegranate, redcurrant, red skinned pear, rosehip, strawberry, etc.), vegetables (asparagus, eggplant, chili peppers, red cabbage, purple cabbage, purple carrot, purple cauliflower, purple turnip, red onion, red radish, purple sweet potato, red turnip, tomatoes, violet peppers, etc.), legumes (black bean, black soybean, etc.), and cereal grains (black rice, black-purple rice, black sorghum, purple barley, red sorghum, rye, etc.) [21], which are consumed by humans, and hence anthocyanins are considered as the most common beneficial components of the human diet due to their antioxidant properties. Anthocyanins are used in traditional medicines and scientific evidence proved that they are potent pharmacological constituents, attributed to their ability to nullify oxidative stress [20,22]. The current manuscript summarizes the recent reports on the influence of supplementation of anthocyanins on obesity-associated comorbidities. The scientific information has been retrieved from Web of Science, PubMed, Scopus, and Google Scholar using the search terms “anthocyanin” and “obesity”. Documents that are published in English have been used without any chronological restrictions for the preparation of the current manuscript.

## 2. Anthocyanins and Obesity

### 2.1. Outcomes of Human Clinical Studies 

Bertoia et al. [23] conducted three cohort studies and evaluated the effect of consuming dietary flavonoids including anthocyanins, flavanols, flavones, flavanones, and flavonoid polymers on weight management in healthy female nurses (daily median intake of 247 mg of total flavonoids) aged between 30–55 years (study initiated in 1976), male health professionals (daily median intake of 224 mg of total flavonoids) aged between 40–75 years (study initiated in 1986), and female nurses (daily median intake of 236 mg of total flavonoids) aged between 25–42 years (study initiated in 1989). The dietary information of the participants was collected by a semiquantitative food frequency questionnaire every four years. A questionnaire was used to self-report the weight of the participants every two years, and changes in weight over each four-year duration were calculated between 1986 and 2011. The results of the cohort study suggested that the positive benefits of dietary flavonoids consumption are closely associated with anthocyanins and pro-anthocyanidins. The anthocyanins and a pro-anthocyanidins-rich diet help to maintain body weight and prevent obesity and its associated consequences in healthy adults [23]. Daily intake of anthocyanins and pro-anthocyanidin rich fruits and vegetables might aid weight maintenance in every healthy individual.

Cyanidin-3-*O*-β-D-glucoside and cyanidin-3-(6”-malonyl)-β-glucoside were found to be the major anthocyanins among those identified in red orange juice of Morro cultivar [24]. Silveira et al. [25] examined the effect of consuming ready-to-drink red-fleshed sweet orange juice on the risk factors involved in the development of metabolic syndrome in overweight or obese individuals and normal individuals. After eight weeks of daily intake of red orange juice (750 mL of orange juice per day), significant increase in serum antioxidant activity and reduction in the levels of C-reactive protein, TC (total cholesterol), and LDL-C (low-density lipoproteins-cholesterol) were observed in individuals of both normal weight and overweight/obese groups, along with significant reduction of diastolic blood pressure observed in overweight or obese individuals, while systolic blood pressure, fasting insulin levels, and insulin resistance were significantly reduced in normal weight individuals. Consumption of ready-to-drink red-fleshed sweet orange juice might prevent the development of metabolic syndrome by exhibiting beneficial effects such as improved lipid profile, antioxidant and anti-inflammatory activity [25]. Azzini et al. [26] determined the effect of daily intake of commercial red orange juice on body composition and metabolic status in overweight or obese women. Daily intake of commercial orange juice (Dosage, 500 mL of orange juice per day; i.e., 250 mg of anthocyanins per day) rich in anthocyanins (>50 mg of anthocyanins per 100 mL of orange juice as per the data fixed by the commercial brand Ortogel, Catania, Italy) for 12 weeks showed no significant alteration in body weight, BMI, waist and hip circumferences, and waist/hip circumferences in obese female subjects. The level of TC and LDL-C was reduced after orange juice consumption when compared to baseline levels, whereas biochemical and hemodynamic parameters were not affected significantly upon orange juice consumption. Inflammatory and metabolic biomarkers including adiponectin (non-significant decrease after the supplement phase, and after one month follow-up), TNF-α, c-reactive protein, leptin, insulin, glucose and HOMA-IR (Homeostatic Model Assessment-Insulin resistance) were not improved after the consumption of orange juice and at follow-up when compared to baseline. Consumption of red orange juice showed reduction in TNF-α and c-reactive protein in only two overweight individuals. Therefore, consumption of commercial orange juice showed an improvement in the lipid profile (TC and LDL-C levels) and no beneficial effect on obesity, inflammatory status, and insulin resistance [26]. Scordino et al. [27] reported that only 60% of the tested commercial blood orange juice contains the minimum anthocyanin content labeled by the brand. Anthocyanin content of commercial orange juices might degrade due to improper storage conditions [27], which might contribute the lack of or reduced beneficial effects of commercial orange juices. Choosing the brand that meets quality and retains active content during the shelf-life duration that is labeled on the product is important to obtain the health benefits of the orange juices. Thus, consumption of fruits or fresh juices is a cheaper and better option to ensure the intake of health beneficial components available in fruits rather than consuming stored commercial products.

Hester et al. [28] conducted an open-labelled study and investigated the beneficial effect of an anthocyanin-prebiotic blend on the intestinal microbiota, intestinal inflammation, and bowel habits in adult individuals with uncomplicated obesity. Daily supplementation of an anthocyanins blend (total of 215 mg anthocyanins per day: extracts of European blueberry, black rice, and blackcurrant) and prebiotic fiber (total of 2.7 g per day: Inulin and fructooligosaccharides) consumed with breakfast for eight weeks significantly improved abdominal pain, bloating severity, and stool consistency in individuals with uncomplicated obesity. Fecal firmicutes and actinobacteria load were reduced and Bacteroidetes content was increased after the intervention. Body mass and BMI were not changed during and after the intervention. HbA1c level was decreased significantly while inflammatory fecal calprotectin reduction was not significant compared to baseline values. The results suggested that the regular consumption of anthocyanins and prebiotic blend could significantly improve the intestinal environment via positive regulation of microbiota in individuals with uncomplicated obesity [28].

Blueberries are known for their high content of anthocyanins. The major anthocyanins of highbush blueberries include 3-glucosides, 3-arabinosides and 3-galactosides of delphinidin, malvidin, and petunidin [21]. Istek and Gurbuz [29] studied the impact of consuming highbush organic blueberries on the body weight and metabolism of obese individuals during 12 weeks of nutrition therapy. After first six weeks of nutrition therapy, one serving of fruit (50 g of carbohydrate source) in the diet was replaced with blueberries (blueberries are rich in anthocyanins; 50 g per day) in the blueberry group. The changes in body weight, body fat, cholesterol profile, and metabolic biomarkers were recorded. The results showed that the blueberries-supplemented group exhibited a significant level of weight reduction and body fat reduction. The TC, LDL-C levels, insulin, insulin resistance, and uric acid significantly improved in the blueberry group when compared to baseline and placebo control. Notably, 2.5 to 3-fold of BMI reduction was observed in the blueberry-supplemented group at the end of 12 weeks, which was a significant health impact. The positive health benefits of blueberries might be attributed to the high content of anthocyanins and phenolic compounds [29].

Queen Garnet (QG) plum juices are rich in cyanidin 3-*O*-β-D-glucoside (C3G). The influence of consumption of QG juice on cardiovascular and metabolic disease risk in mildly hypertensive overweight/obese individuals was investigated by Bhaswant et al. [30]. Twelve-week consumption of QG juice (250 mL juice per day; 102 mg C3G equivalents of total anthocyanins, and 36 mg of quercetin glycosides per 100 mL of QC juice) significantly reduced the blood pressure, fasting plasma LDL, glucose, insulin, C-peptide, GLP-1 (Glucagon-like peptide-1) and leptin levels, and IL-2, IL-6, IL-13, and TNF-α concentrations, while no changes in HDL (High-density lipoproteins), GGT (γ-glutamyl transferase), and glucagon were found in obese subjects. The adiponectin content was increased in the QG juice group. There were no changes in TC, TG (triglycerides), ALT (Alanine transaminase), AST (Aspartate transaminase), PAI-1 (Plasminogen activator inhibitor-1), and creatinine in QG juice interventions. The overall study suggested that the consumption of QG juice reduced the blood pressure and risk factors associated with metabolic disorders, but showed no positive signs of obesity reversal [30].

Juҫara berry is rich in anthocyanins and other phenolic compounds. The major anthocyanins include C3G and cyanidin 3-rutinoside. The minor anthocyanin content includes cyanidin-3,5-diglucoside, peonidin-3-glucoside, peonidin-3-rutinoside, and pelargonidin-3-rutinoside) [31]. Santamarina et al. [32] conducted a randomized, double blind, placebo controlled trial and evaluated the effect of consuming freeze dried juҫara berry (five g per day for six weeks) on inflammatory status of monocytes from obese individuals. The blood monocytes were collected from the experimental subjects at the beginning and end of the study, stimulated with lipopolysaccharides, and the regulation of inflammatory biomarkers’ were analyzed. Juҫara berry supplementation reduced the TLR4, IL-6, MYD88, TNF-α, and MCP-1 level, and increased the Ob-R protein, and IL-10. Jucara berry consumption effectively diminished the pro-inflammatory markers in obese subjects, which indicated that Juҫara pulp might control the inflammatory status of obesity [32].

The supplementation of purified anthocyanins (320 mg per 4 capsules per day taken along with usual diet; each capsule contains 3-O-rutinoside of cyanidin and delphinidin, and 3-O-β-galactosides, 3-O-β-glucosides and 3-O-β-arabinosides of cyanidin, delphinidin, malvidin, petunidin and peonidin) of blackcurrant and bilberries for 28 days significantly reduced the plasma CCL2 (C-C Motif Chemokine Ligand 2) level in obese, overweight and lean individuals. The IL-6 level was significantly reduced in obese subjects after anthocyanins interventions. The hematological, biochemical, and lipid profiles were not significantly altered by anthocyanins intervention. The results recommended that the regular intake of anthocyanins reduced obesity-associated inflammation in overweight/obese subjects [33] (Table 1).

### 2.2. Results of In Vivo Studies 

The supplementation of anthocyanin-rich Norton grape pomace extract (250 mg/kg of body weight/day) for 12 weeks reduced the plasma C-reactive protein level in high-fat-diet-induced (93.5 g protein, 106.2 g fat, 240.9 g carbohydrate are the macronutrient content of the diet per 10 megajoules) obese mice. The antioxidant capacity of the mice was not improved by grape extract intervention. There was no significant change in body weight of experimental mice supplemented with grape extract. The results showed that Norton grape pomace extract supplementation exhibited anti-inflammatory effects in high-fat-diet-induced obese mice [34].

Mulberry water extracts (MWE) are rich in polyphenols including anthocyanins. The dietary supplementation of different concentrations of MWE (0.5–2.0% w/w) significantly suppressed the high-fat diet-induced obesity in the hamster model via hypolipidemic activity. The hamsters co-supplemented with a high-fat diet (0.2% cholesterol and 10% corn oil were added in chow as high fat diet) and MWE exhibited a reduction in body weight and visceral fat mass. The MWE supplementation reduced the serum triacylglycerol, total cholesterol, LDL/HDL ratio, free fatty acids, and hepatic lipids value. The hepatic expression of carnitine palmitoyltransferase-1 and peroxisome proliferator-activated receptor α were induced while 3-hydroxy-3-methylglutaryl-coenzyme A (HMG-CoA) reductase and fatty acid synthase expressions were hindered. The results hypothesized that MWE regulates lipogenesis and lipolysis in the high fat diet-induced experimental obese hamster model, and MWE might be considered as a supplementary therapeutic agent to manage obesity [35].

The supplementation of freeze-dried whole highbush blueberry powder (2% wt/wt) in high-fat-diet-fed (45% of kcal) male Zucker rats for 90 days showed anti-obesity activity. Blueberry powder intervention reduced TG, fasting insulin, HOMA-IR, glucose area under the curve, and abdominal fat mass. Further, the blueberry supplemented group showed an increase in peroxisome proliferator-activated receptor (PPAR) associated with adipose and skeletal muscle, and the regulatory role of PPAR related to the fat oxidation and glucose uptake and oxidation was also affected, which suggested that the blueberry ingestion stimulated fat and glucose metabolism effectively, and thereby exhibiting anti-obese activity [36].

Likely the supplementation of blueberry and mulberry juice [37] or mulberry anthocyanins (40 or 200 mg**/**kg) [38] for 12 weeks significantly suppressed body weight gain and reduced insulin resistance and lipid accumulation in high-fat-diet-fed (45% calories from fat) mice. Moreover, the intervention decreased serum cholesterol and leptin secretion in obese mice. The results showed that anthocyanin-rich berries may effectively hinder diet-induced obesity [37,38].

The supplementation of pure anthocyanins (C3G; 1g/kg of body weight) for 12 weeks prevented the incidence of obesity in KK-*Ay* mice (the diabetic KK strain was originally established by Professor Kyoji Kondo (1957); KK-*Ay* mice are a cross between diabetic KK and lethal yellow A^y^ (agouti yellow) mice). The anthocyanins supplemented animals showed a reduction in weight gain, and reduced liver and visceral adipose tissue weight compared to control. The level of TG in plasma and liver was reduced in C3G-treated animals. The steatosis score was significantly reduced in the C3G-treated group. The pAMPK (adenosine monophosphate-activated protein kinase phosphorylation) was increased in skeletal muscle and visceral adipose tissues, which further stimulated lipoprotein lipase (LPL) activity in skeletal muscle and plasma, but LPL activity was not observed in visceral adipose tissues. The results suggested that the intervention of C3G controls obesity via LPL activation [39].

The intervention of purple sweet potato-anthocyanins (200 mg of anthocyanin fraction/kg body weight/day) for four weeks reduced body weight gain, TG, and TC levels in high-fat diet-induced (45% calories from fat) obese mice. The anthocyanins supplementation significantly reduced TBARSs (thiobarbituric acid reactive substances) content and hepatic lipid accumulation. Anthocyanin increased pAMPK and pACC (Acetyl-coenzyme A carboxylase phosphorylation) activity and suppressed sterol regulatory element-binding protein 1 and its associated players including fatty acid synthase. The inhibition of phosphorylation of AMPK may prevent the beneficial effects of anthocyanins, which indicated that AMPK signaling plays a key role in anthocyanins-mediated anti-obesity activity [40].

C3G-rich purple corn color significantly suppressed high-fat-diet-induced weight gain and adipose tissue weight in mice model. Anthocyanin effectively protects the mice from high-fat-diet-induced hyperleptinemia, hyperglycemia, and hyperinsulinemia. The protective nature of the anthocyanin was attributed to its ability to regulate the genes involved in fatty acid and triacylglycerol synthesis. The results suggested that C3G can prevent the high-fat-diet-induced obese condition in mice [41]. Tomay et al. [42] reported that the supplementation of purple corn extract (290 mg of anthocyanins equivalent/kg/day) positively regulates inflammatory mediators, M2 markers, iron metabolism-related genes, and NF-kB signaling. Treatment also enhanced lipid metabolism and reduced inflammatory reaction in high-fat diet-induced obese mice. The results proved that purple corn extract amended high-fat-diet-induced inflammation and adipose tissue phenotype in mice [42].

The supplementation of anthocyanins-rich chokeberry extract (CBE) protects the experimental animal from fructose-rich diet-induced health complications. CBE supplementation (100 or 200 mg/kg body weight**/**day for 6 weeks) reduced epididymal fat, TC, LDL-C, and blood glucose level in the rat. The elevated plasma adiponectin was observed in CBE treated group while TNF-α, IL-6, inflammatory cytokines levels, and mRNA expressions of *Fabp4, Fas*, and *Lpl* were reduced. The CBE supplementation significantly induced the mRNA expressions of *Gys1, Irs1, Irs2, Pi3k, Glut1, Glut4*, *Pparγ*, adiponectin, and zinc finger protein in fructose-rich diet-fed rats. The results proposed that CBE has the potential to prevent diet-induced obesity-associated insulin resistance, inflammation and adipogenesis [43].

Badshah et al. [44] demonstrated that the supplementation of anthocyanins of black soybean seed coat has anti-obesity potential in the Sprague-Dawley rat model. The administration of anthocyanins (6 or 24 mg/kg for 40 days) significantly slowed down bodyweight gaining and food consumption in Sprague-Dawley rats compared to control. The notable amount of reduction in adipose tissue mass was observed in an anthocyanins-treated group, and the reduction was dose-dependent. Further, the elevated expression of GABA (γ-aminobutyric acid)-receptor (GABA_B1_R), and down-regulation of neuropeptide Y, PKA (protein kinase A-α), and p-CREB (phosphorylated cAMP-response element-binding protein) in the hypothalamus were attributed to the attenuation of weight gaining of rats in the treatment group. The study proved that anthocyanins regulate food consumption and weight gaining via neuropeptide Y and GABA_B1_R.

The supplementation of anthocyanins rich grape-bilberry juice (1551 mg of anthocyanins/L) for 10 weeks significantly reduced serum level cholesterol, TG, leptin, and resistin while glucose, insulin, and non-esterified fatty acids level were not changed in Fischer rats. Further, anthocyanin supplementation declined saturated fatty acids and increased polyunsaturated fatty acids levels in rats. There was no change in serum adiponectin and adipokines secretion during the intervention. The results indicated that the consumption of anthocyanins rich grape-bilberry juice precludes the fat-diet induced (34% calories from fat with high content of saturated fatty acids) obesity-associated consequences by improving the plasma fatty acid profile [45].

The supplementation of 1.5% blackcurrant pomace (BP) for four weeks significantly nullified the high-fat-diet-induced (7% and 32% calories from fat) consequences in New Zealand white rabbits. Specifically, BP supplementation reduced putrefactive metabolites and β-glucuronidase activity, and serum level of TG, TC, LDL-C and free fatty acid content in experimental animals. The antioxidant capacity of the host was increased after BP intervention. The results claimed that polyphenol-rich BP could suppress the diet-induced obese condition [46].

The high-fat diet (45% calories from fat) and black soybean testa (BST; 1g/kg in body weight) supplementation significantly reduced food consumption, fat accumulation, and expression of acetyl-CoA carboxylase and CCAAT-enhancer-binding protein-α in mice, whereas, the expression of AMP-activated protein kinase, hormone-sensitive lipase, and lipoprotein lipase were increased after BST intervention. BST supplementation positively regulated TNF-α, IL-6 and IL-10 in high-fat diet-fed mice compared to controls. The results suggested that BST supplementation improved the inflammatory system, lipolysis, and lipogenesis in high-fat-diet-fed mice and prevents obesity [47].

Anthocyanins-rich Aronia fruit extract (AFE) supplementation (0.4% anthocyanins in the diet for 4 weeks) effectively suppressed the accumulation of visceral fat, and also reduced perirenal, mesenteric, epididymal adipose tissue mass in high-fat-diet-fed rats when compared to control and bilberry-supplemented group. AFE supplementation inhibited the TG level and blood glucose level in experimental rats during oral corn oil and sucrose tolerance test, respectively. The results proposed that AFE supplementation could improve the fat composition and glycemic conditions of high-fat-diet-fed rats [48].

The supplementation of bilberry extract (0.1% w/w containing 36% of anthocyanins) for 24 weeks did not significantly reduced the diet-induced TC increase and hepatic lipid content in mice, whereas, the expression of *Tnf* genes and the circulation of neutrophil chemoattractant mKC were reduced in high-fat diet (45% kcal fat) and bilberry extract supplemented group compared to control. The study suggested that bilberry extract may secure the mice from inflammatory damage associated with diet-induced obesity [49]. Peixoto et al. [50] reported that the intervention of cranberry extract significantly reduced weight gain, hepatic cholesterol level, lipid peroxidation, protein carbonylation, fatty acid synthase level, and fat accumulation in high-fat-diet (44.5% calories from fat) fed rats.

The anti-obesity property of blueberry anthocyanins was demonstrated in high-fat diet (45% calories from fat) supplemented mice [51]. The supplementation of a high concentration of blueberry anthocyanins (200 mg/kg) significantly reduced the weight gain in high-fat diet supplemented mice. The expression of TNF-α, IL-6, PPARγ, and FAS genes was reduced. The serum glucose level and epididymal adipocytes were reduced in the experimental mice. TC, TG, and lipids content were reduced in blueberry anthocyanins supplemented diet-induced obese mice. The results suggested that the supplementation of a high concentration of blueberry anthocyanins might protect the mice from diet-induced obesity-related consequences [51]. Likely, the supplementation of mulberry and cherry anthocyanins (200 mg/kg) prevented weight gain and nullified oxidative damages in high-fat-diet-induced (45% calories from fat) obese mice. Specifically, SOD (Superoxide dismutase) and GPx (Glutathione peroxidase) activities were improved, and the expression of TNF-α, IL-6, iNOS, and NF-кB genes were suppressed in anthocyanins supplemented group compared to control. The high-fat-diet-induced distortion in the lipid profile was amended in the anthocyanins supplemented group. The results supported that mulberry and cherry anthocyanins improve the inflammatory and antioxidant system and prevent weight gain in high-fat-diet-induced obese mice [52]. The supplementation of 10% blueberry powder along with high-fat diet (45% calories from fat) improved insulin sensitivity, altered the microbiota (increased the abundance of *Gammaproteobacteria*), and normalized the ileal villus height and TNF-α and IL-1β expressions in rats compared to control. The results suggested that blueberry intervention improved insulin signaling, inflammation and gut microbiota [53]. Skates et al. [54] reported that the consumption of malvidin, petunidin, and peonidin rich berry improved body composition via enhancing energy expenditure, amended mitochondrial respiration and reduced metabolic stress in the high-fat-diet-fed C57BL/6 mouse.

The supplementation of *Acanthopanax senticosus* fruits (0.5 or 1.0 g/kg) for 12 weeks improved insulin resistance and hepatic lipid accumulation in high-fat-diet-fed (20.3% calories from fat) mice. The expression of FAS was reduced, and the expression of cholesterol 7-α-hydroxylase was induced after *A. senticosus* fruit supplementation in high-fat-diet-fed mice. The phosphorylation of AMPK was increased and the expression of genes involved in the lipid metabolism was improved in *A. senticosus* fruits supplemented group compared to control. The high concentration of *A. senticosus* fruits supplementation showed higher benefits compared to that of the low concentration. The results reveal that *A. senticosus* fruits rich in anthocyanins protect high-fat-diet-fed obese mice from dyslipidemia and insulin resistance via pAMPK and lipogenic gene regulation [55].

The supplementation of sweet cherry anthocyanins (200 mg/kg for 15 weeks) alter the expression of hepatic genes and protects the high-fat-diet-fed mice from hepatic steatosis via PPARγ pathway [56]. The mulberry ethanol extract (rich in C3G and cyanidin-3-rutinoside) intervention significantly inhibited the high-fat diet-induced weight gain. The mulberry ethanol extract improved the hepatic steatosis and adipose hypertrophy in diet-induced obese mice via regulating the expression of genes involved in lipid and cholesterol metabolism. Moreover, mulberry ethanol extract intervention improved insulin resistance and glucose hemostasis in high-fat-diet-induced obese mice [57].

The supplementation of anthocyanin rich purple potatoes improved blood pressure, glucose tolerance, and insulin sensitivity in high-fat-diet-fed Zucker rats [58].

The intervention of *Vitis vinifera* L. grape skin extract (200 mg/kg**/**day) for 12 weeks improved the health status of high-fat-diet-fed (60% calories from fat) mice. *Vitis vinifera* L. grape skin extract prohibited body weight gain, insulin resistance and dyslipidemia, and maintained the leptin and adiponectin levels in plasma and adipose tissue. The regulation of insulin signaling players GLUT4, pAMPK/AMPK ratio is the possible reason behind the *Vitis vinifera* L. grape skin extract mediated beneficial effect. Moreover, *Vitis vinifera* L. grape skin extract supplementation improved the antioxidant state of the mice, especially decreasing the MDA and carbonyl levels in adipose and muscle tissues. The levels of inflammatory markers were also suppressed in *Vitis vinifera* L. grape skin extract-treated mice. Overall, the results revealed that the supplementation of *Vitis vinifera* L. grape skin extract could improve high-fat-diet-induced obesity and metabolic disorders in mice [59].

Red raspberry fruit supplementation did not affect weight and body fat mass while oxidative stress level was reduced through increasing GPx activity and reducing the reactive oxygen species (ROS) generation of DNA damage in obese diabetic mice. The activities of SOD and catalase (CAT) were not changed in the control and treatment groups during the experiment. The plasma IL-6 concentration was low in the raspberry group compared to control. The lipid peroxide, TG, and TC levels were not affected by the intervention. The results showed that raspberry fruit intervention protects the mice from diabetes-induced oxidative stress [60].

The supplementation of purple sweet potato color (PSPC) at a concentration of 700 mg/kg/day for 20 weeks suppressed the development of obesity and liver damages in high-fat-diet-fed (60% calories from fat) mice. PSPC intervention suppressed the hepatic oxidative stress and restored the NAD^+^ level to decrease endoplasmic reticulum stress in high-fat-diet-fed mice. The nucleotide oligomerization domain protein1/2 signaling, nuclear factor- κB (NF-κB) p65 nuclear translocation were suppressed in PSPC-treated mice. PSPC intervention reduced the inflammasome activation, thereby diminishing the inflammation-associated genes in high-fat diet-fed mice. The results suggested that PSPC supplementation protected the mice from high-fat-diet-induced hepatic inflammation through NAD^+^ and inhibition of NLRP3 (NOD-, LRR- and pyrin domain-containing protein 3) inflammasome activation [61].

The intervention of 1 mg/mL of C3G for 16 weeks effectively exhibited anti-obesity activity, which was witnessed by the increase in energy expenditure, suppression of weight gain, normalization of hepatic steatosis, glucose homeostasis, and cold tolerance in obese db/db mice. The brown-like adipocytes formation was induced in C3G treated mice compared to control. C3G supplementation regulated the expression of uncoupling protein 1 (UCP1) in db/db mice. The results claimed that the C3G could be a potent therapeutic agent to treat obesity [62]. Similarly, C3G supplementation positively altered the thermogenic status, energy expenditure, and expression of UCP1 in high-fructose and high-fat-diet-fed mice [63].

The intervention of anthocyanins of blueberry (BBA) and blackberry (BLA) (200 mg of BBA or BLA/kg) for 12 weeks improved the oxidative stress and inflammation and increased the energy expenditure in high-fat-diet-fed (45% calories from fat) obese mice. The serum level of TG, TC, LDL-C, and MDA was reduced, and GPx activity was increased in anthocyanins supplemented mice compared to control. BLA supplementation increased the HDL-C level without affecting the SOD and leptin, whereas BBA affected the leptin and SOD but not HDL-C. The anthocyanin treatment significantly reduced hepatic lipid and MDA levels and induced SOD and GPx activities compared to control. BBA supplementation effectively reduced hepatic TG level and increased GPx activity compared to BLA-treated mice. Anthocyanins treatment affected the fecal short-chain fatty acid (SCFs) content; particularly, the concentrations of acetic, propionic, butyric, and valeric acids were increased while isobutyric acid was reduced. The expression of IL-6, TNF-α, and NF-κB was reduced significantly in the anthocyanins-treated group. The metabolism of glycerophospholipid and glutathione and the insulin-signaling pathway were affected by the BLA and BAA supplementation. The results suggested that the intervention of BAA and BLA improved high-fat-diet-induced inflammation, and oxidative damages through enhancing energy expenditure [64]. Shi et al. [65] reported that intervention of blueberry or C3G or in combination improved glucose tolerance and reduced total body mass, body fat, and blood pressure in high fructose (30%) and high-fat-diet-fed (59% calories from fat) mice. 

The supplementation of anthocyanins mix, rich in cyanidin and delphinidin, significantly diminished high-fat-diet-induced (60% calories from fat) obesity, dyslipidemia, insulin resistance, inflammation, and liver lipid deposition in mice. Anthocyanins supplementation normalized the level of monocyte chemoattractant protein-1 (MCP-1), TNFα, F4/80 (macrophage marker), and inducible nitric oxide synthase (NOS2) in the liver of mice. The diet-induced NADPH oxidases, oxidized lipid-protein adducts, and H_2_O_2_ were normalized in the anthocyanins-treated group, and also the activation of JNK1/2, IKK/NF-κB signaling and protein tyrosine phosphatase 1B (PTP1B) expression were suppressed. The study proved that the consumption of an anthocyanins rich diet helps to ameliorate the consequences of obesity and metabolic disorders [66]. Marques et al. [67] proved that the supplementation of anthocyanin-rich blackberry extract effectively improved high-fat-diet-induced dysbiosis. Moreover, the alteration in the gut microbiome was linked with an anti-neuro inflammatory response against diet-induced obese conditions. Additionally, tryptophan metabolism and neuroprotective metabolite level were improved in the anthocyanins supplemented group compared to control. The study strongly sustained that anthocyanin-rich blackberry extract could positively regulate gut microbiome and exhibits protection against diet-induced neuro damages in the rat.

The supplementation of high tannin-containing *Plinia jaboticaba* (Vell.) Berg extract suppressed high-fat-sucrose diet-induced weight gain, dyslipidemia, hyperglycemia, and insulin resistance in C57BL/6 mice. The hike in TC level was attenuated in high tannin-containing *Plinia jaboticaba* (Vell.) Berg extract-treated mice. The beneficial effect was attributed to the richness of phenolic compounds, especially anthocyanins. The intervention of low tannin-containing extract did not show significant beneficial effects compared to high tannin-containing extract. The results showed that high tannin-containing *Plinia jaboticaba* (Vell.) Berg extract could be a potent anti-obesity agent [68].

A study by Cremonini et al. [69] exposed that intervention anthocyanins (40 mg/kg) for 14 weeks significantly mitigate high-fat-diet-induced (60% calories from fat) intestinal disintegration, oxidative stress, and dysbiosis. Specifically, anthocyanin supplementation regularized the expression of claudin-1, occludin, and ZO-1, GLP-2 (Glucagon-like peptide-2), MUC2 (Mucin 2), NOX1 (NADPH Oxidase 1), NOX4 (NADPH Oxidase 4) and NOS2, and redox signaling. The damaged microbial composition has been restored by anthocyanins in high-fat-diet-fed mice. Anthocyanins, particularly delphinidin and cyanidin, supplementation might improve high-fat-diet-induced obesity-related consequences. 

The supplementation of Davidson’s plum (8 mg of anthocyanins equivalent/kg/day) for 8 weeks improved metabolic disorders in high-carbohydrate and high-fat diet-fed rats. Davidson’s plum supplementation effectively reduced plasma TG, visceral fat accumulation, abdominal fat mass, retroperitoneal adipocytes mass, fatty acid content, and inflammatory infiltrations and decreased the collagen deposition and fat vacuoles in heart and liver, respectively. The degeneration of knee cartilage was reduced in Davidson’s plum supplemented group with no change in glucose tolerance. The fecal microbiota analysis showed that Davidson’s plum treatment altered the abundance of *Turicibacter* spp., Clostridiaceae spp. and *Akkermansia muciniphila.* The results suggested that the consumption of anthocyanin-rich Davidson’s plum could improve diet-induced metabolic syndrome [70].

Lim et al. [71] revealed that the supplementation of *Aronia melanocarpa* extract, rich in C3G, prevented weight gain and fat accumulation in high-fat-diet-fed (60% energy from fat) mice in a dose-dependent manner. *A. melanocarpa* extract supplementation suppressed the adipogenesis by regulating the expression of genes involved in adipogenesis. The results showed that *A. melanocarpa* extract could suppress high-fat-diet-induced obesity.

Nemes et al. [72] found that the intervention of anthocyanin-rich tart cherry extract effectively improved the antioxidant capacity of high-fat-diet-fed (45% energy from fat in diet and 5% sucrose in water) mice, but failed to retain body mass and glucose tolerance. Levels of IL-6 and leptin were reduced in the treatment group compared to control. The results showed that tart cherry extract could develop the radical scavenging capacity of the host and improve the inflammatory markers related to obesity. 

The intervention of Maqui berry, rich in delphinidin-3-*O*-sambubioside-5-*O*-glucoside and delphinidin-3-*O*-sambubioside, for 16 weeks (20 mg/mL in water per day) ameliorates high-fat-diet-induced (45% energy from fat) insulin resistance. Moreover, the treatment suppressed weight gain and affected the regulation of genes involved in fatty acid oxidation, lipogenesis, thermogenesis, and multilocular lipid droplet formation in subcutaneous white adipose tissue, which were attributed to the change in expression of *Chrebpb* (carbohydrate response element binding protein b), *Creg1* (cellular repressor of adenovirus early region 1A-stimulated genes 1), and *Srebp1c* (sterol regulatory binding protein 1c). The improvement in fibroblast growth factor 21 signaling was also involved in the beneficial effects of Maqui berry supplementation. The results found that the intervention of Maqui berry could improve high-fat-diet-induced obesity and associated illnesses [73] (Table 2).

### 2.3. Results of in vitro Studies 

Anthocyanins (cyanidin or C3G) treatment (100 µM) improved the secretion of adiponectin and leptin in rat adipocytes and increased the expression of adipocyte-specific genes without activating PPARγ. Specifically, cyanidin treatment increased the leptin secretion but C3G treatment did not affect the secretion of adipocytokine [74]. Cyanidin or C3G suppressed the inflammatory responses, and MCP-1 and MRP-2 activation and release in 3T3-L1 adipocytes in a dose-dependent manner [75].

C3G treatment suppressed glycerol and FFAs release in 3T3-L1 adipocytes in a time and dose-dependent manner. The hexosamine biosynthetic pathway was suppressed by C3G treatment in adipocytes, which was characterized by the changes in the activities of AMP-activated protein kinase, glutamine: fructose 6-phosphate aminotransferase, and reduction of cellular UDP-N-acetylglucosamine production. Further, C3G treatment diminished the *O*-glycosylation of FoxO1 and suppressed the expression of adipose triglyceride lipase. The results suggested that C3G treatment could inhibit adipocyte lipolysis via FoxO1-mediated adipose triglyceride lipase expression [76].

Grape anthocyanin treatment significantly reduced the accumulation of TG in 3T3-L1 adipocytes in a dose-dependent manner. The expression of lipogenic transcription factors was positively regulated in anthocyanins treated adipocytes. Particularly, expressions of LXRα (Liver X receptor α), SREBP-1c (Sterol regulatory element-binding protein-1c), PPARγ (peroxisome proliferator-activated receptor γ), C/EBPα (CCAAT/enhancer-binding protein α), FAS (fatty acid synthase), SCD-1 (Stearoyl-CoA desaturase-1) and ACCα (Acetyl-CoA carboxylase α) were suppressed. The results showed that grape anthocyanin treatment reduced lipid accumulation by regulating the genes involved in lipogenesis [77]. Anthocyanins from *Vitis coignetiae* Pulliat (AVC) suppressed the differentiation of adipocytes, reducing the lipid and TG accumulation in 3T3-L1 adipocytes. AVC treatment activated the AMP-activated protein kinase and suppressed the expressions of PPARγ, C/EBPα, SREBP-1c, FAS, leptin, and adipocyte fatty acid-binding protein. The results revealed that AVC has anti-obesity potential, which was attributed to the ability of inhibition of adipogenesis and adipocyte differentiation [78]. Likely, delphinidin-3-*O*-β-glucoside treatment inhibited the accumulation of lipids and downregulated the expressions of PPARγ, C/EBPα, SREBP-1c, and FAS; whereas, the expression of SIRT1 (silent mating type information regulation 2 homolog 1) and CPT-1 (carnitine palmitoyltransferase-1) were upregulated in 3T3-L1 adipocytes. Moreover, delphinidin-3-*O*-β-glucoside treatment activated the phosphorylation of AMPK and acetyl-CoA carboxylase (ACC), which suggested that delphinidin-3-*O*-β-glucoside could suppress adipogenesis and promoted lipid metabolism [79].

The purple corn silk extract (PCS) rich in anthocyanins exhibited anti-obesity activity in vitro. At the concentration of 250–1000 μg/mL, PCS significantly suppressed the preadipocyte proliferation (up to 75.51%) and decreased the total lipid accumulation (up to 69.62%). PCS treatment increased the release of glycerol and induced apoptosis, which was characterized by apoptotic bodies and condensed nuclei in the 3T3-L1 cell line [80].

Raspberry polyphenolic extract (RPE) modified hepatic immune-metabolic mechanisms in hepatocytes via regulating the expressions of CD44, STAT1, ANGPTL4, COX-2, and AhR. The endosomal/lysosomal activity was not restored by RPE in hepatocytes. The study claimed that RPE could regulate the obesity-associated singling pathways in hepatocytes and have anti-obesity potential [81]. 

The anti-obesity activity of anthocyanins from *Cornus kousa* (ACK) has been demonstrated in HUVEc (human umbilical vein endothelial cell) and 3T3-L1 cells. ACK treatment significantly suppressed the differentiation of adipocytes and the accumulation of lipids in a dose-dependent manner via downregulating NF-kB, PI3K, vascular endothelial growth factor 2 (VEGRF 2), β-catenin, and Akt1. The expression of PPARγ, CCAAT, C/EBPα, aP2 (fatty acid-binding protein), FAS, and lipoprotein lipase (LPL) were downregulated after ACK treatment in 3T3-L1 cells. ACK treatment inhibited lipogenesis and adipogenesis by regulating the respective genes. The study claimed that anthocyanins-rich *C. kousa* extract could be used as an anti-obesity agent [82].

Anthocyanins treatment suppressed lipid accumulation, PPAR-γ level, and NF-κB activation in palmitic acid-induced 3T3-L1 adipocytes in a dose-dependent manner. Moreover, anthocyanins treatment improved insulin sensitivity and suppressed the comorbidities of obesity in vitro [83]. Cyanidin and C3G-rich ethanolic extract of *Vigna unguiculata* showed significant pancreatic lipase inhibition in vitro, which indicated that *V. unguiculata* anthocyanins have anti-obesity potential [84] (Table 3).

## 3. Conclusions

Several in vitro and in vivo studies confirmed that the consumption of anthocyanins rich food prevents or treats obesity-associated consequences like diabetes, inflammation and oxidative stress. However, very limited studies have been performed on human volunteers. The results suggested that daily intake of anthocyanin rich (5.7 to 11.5 mg per day) fruits and vegetables might aid weight maintenance in healthy individuals [23], and aid in maintaining or reducing the body weight of obese subjects [29], and improving fatty acid and lipid metabolism [25,26,29,30]; Juҫara pulp might control the inflammatory status of obesity [32]; Queen garnet plum juice (250 mL juice per day; 102 mg C3G equivalents of total anthocyanins, and 36 mg of quercetin glycosides per 100 mL of QC juice) reduced blood pressure and risk factors associated with metabolic disorders in mildly hypersensitive obese individuals [30]; highbush organic blueberries improved the metabolism of obese individuals; daily intake of anthocyanins (total of 215 mg anthocyanins per day; extracts of European blueberry, black rice, and blackcurrant) and prebiotic fiber (total of 2.7 g per day; Inulin and fructooligosaccharides) blend) consumed with breakfast for eight weeks improved gut microbiota in uncomplicated obesity individuals [28]. Though the preclinical studies proved the beneficial effects of anthocyanins, we do not have an established treatment procedure to prevent or manage the over-weight condition and its comorbidities. 

**Figure 2:**
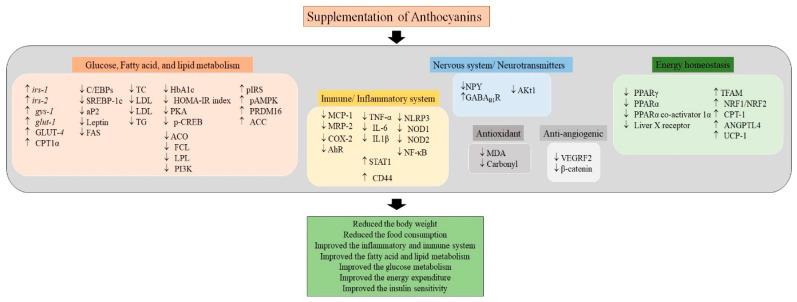
The possible mechanism and the players underlying the anti-obesity associated beneficial effect of anthocyanins. Anthocyanins supplementation positively regulated the genes and proteins involved in glucose, fatty acid, lipid metabolism, energy homeostasis, immune and inflammatory system, antioxidant and anti-angiogenic machineries, and nervous and neurotransmitters, which in turn improved the inflammatory and immune system, fatty acid and lipid metabolism, glucose metabolism, energy expenditure, and insulin sensitivity, and reduced the body weight and food consumption. (ACC: Acetyl-CoA carboxylase; ACO: Acyl CoA oxidase; AhR: Aryl hydrocarbon receptor; AKt1: Serine-threonine protein kinase-1; ANGPTL4: Angiopoietin-like 4; aP2: Adipocyte-specific fatty acid binding protein; C/EBPs: CCAAT/enhancer binding Proteins; COX-2: Cyclooxygenase-2; CPT-1: Carnitine palmitoyltransferase-1; CPT1α: Carnitine palmitoyl-transferase-1α; FAS: Fatty acid synthase; FCL: Fatty acid CoA ligase; GABA_B1_R: Gamma-amino butyric acid receptor; glut-1: Glucose transporter-1; GLUT-4: Glucose transporter-4; gys-1: Glycogen synthase-1; HbA1c: Hemoglobin A1c; HOMA-IR index: Homeostatic Model Assessment for Insulin Resistance; IL-1β: Interleukin-1β; IL-6: Interleukin-6; irs-1: Insulin receptor substrate-1; irs-2: Insulin receptor substrate-2; LDL: Low-density lipoprotein; LPL: Lipoprotein lipase; MCP-1: Monocyte chemoattractant protein-1; MDA: Malondialdehyde; MRP-2: Macrophage inflammatory protein-related protein-2; NF-κB: Nuclear factor κB; NLRP3: NACHT, LRR and PYD domains-containing protein-3; NOD1: Nucleotide-binding oligomerization domain-containing protein-1; NOD2: Nucleotide-binding oligomerization domain-containing protein-2; NPY: Neuropeptide Y; NRF1: Nuclear respiratory factor-1; NRF2: Nuclear respiratory factor-2; pAMPK: Phosphorylated AMP-activated protein kinase; p-CREB: phosphorylated cAMP-response element binding protein; PI3K: Phosphoinositide 3-kinase; pIRS: Phosphorylated insulin receptor substrate; PKA: protein kinase A-α; PPARα: Peroxisome proliferator-activated receptor-α; PPARγ: Peroxisome proliferator-activated receptor-γ; PRDM16: PR domain-containing protei-16; SREBP-1c: Sterol regulatory element binding protein-1c; STAT1: Signal transducer and activator of transcription-1; TC: Total cholesterol; TFAM: Transcription factor A; TG: Triglycerides; TNF-α: Tumor necrosis factor-α; UCP-1: Uncoupling protein-1; VEGRF2: Vascular endothelial growth factor; VLDL: Very-low-density lipoprotein).

As we know, anthocyanins supplementation confers several health benefits to the consumer. As per the literature survey, the anti-obese property of anthocyanins relies on its ability to control food consumption and energy metabolism, and improve inflammatory response, insulin resistance, and glucose metabolism, etc. As represented in Figure 2, anthocyanins supplementation favorably alters the genes and pathways involved in glucose, fatty acid and lipid metabolism, immune and inflammatory system, nervous system, energy homeostasis, antioxidant and anti-angiogenic system. The mechanism behind the favorable effects of anthocyanins was dependent on several physiological, immunological, neurological and metabolic interconnected events.

As mentioned earlier, we are urged to discover the practical application of dietary anthocyanins for the treatment of obesity. Thus, further studies on the optimum dose, duration, and mode of supplementation of anthocyanins are required to develop anthocyanins-based treatment procedures. Well planned clinical studies and post-study follow-ups are necessary to transfer the scientific evidence into a pharmacological application.

## Figures and Tables

**Table 1:** The influence of anthocyanins**/**anthocyanin-rich food supplementation on obesity-associated consequences: outcomes of clinical studies.

Subjects	Study Type	Intervention	Dose & Duration	Results	References
i. Healthy nurses; *n* = 121 701 females; Age = 30–55 years ii. Health professionals; *n* = 51 529 males; Age = 40–75 years iii. Healthy nurses (*n* = 116 686 females); Age = 25–42 years	3 Cohort study (i. Study began in 1976; ii. Study began in 1986; iii. Study began in 1989)	Dietary flavonoids (the major dietary sources include fruits such as apples, blueberries, grapefruit, orange, strawberries; juices such as orange juices, tomato juices; vegetables such as onions, celery, peppers; beverages such as tea, beer, red wine)	Median daily intake of anthocyanins (i. 11.5 mg; ii. 7.7 mg; iii. 5.7 mg), flavonoid polymers (i. mg; ii. mg; iii. mg), flavan-3-ols (i. 24.2 mg; ii. 23.9 mg; iii. 26.1 mg), flavanones (i. 37.6 mg; ii. 41.7 mg; iii. 21.7 mg), flavanols (i. 12.1 mg; ii. 11.7 mg; iii. 14.2 mg), flavones (i. 2.1 mg; ii. 2.2 mg; iii. 1.2 mg); 24 years	Increased flavonoid consumption helps to maintain body weight and prevent the incidence of obesity.	[23]
Overweight/obese individuals; *n* = 12 (3 females, 9 males)/6 (2 females, 4 males); Age = 23–59 years; Average BMI ≥ 25 kg/m^2^ Normal weight individuals; *n* = 17 (11 females, 6 males); Age = 23–59 years; Average BMI = 18.5–24.9 kg/m^2^	Randomized clinical trial	100% ready-to-drink red-fleshed sweet orange juice	750 mL of orange juice per day; 8 weeks	Increased serum antioxidant activity, reduced levels of C-reactive protein, TC, and LDL-C in both groups; reduced diastolic blood pressure in overweight/obese individuals; reduced systolic blood pressure, fasting insulin levels, insulin resistance in normal weight individuals	[25]
Overweight/obese individuals; *n* = 11 females; Age = 36 ± 7 years; Average BMI = 34.4 ± 4.8 kg/m^2^	Pilot Study	Orange juice rich in anthocyanins	500 mL containing 250 mg of anthocyanins per day; 12 weeks.	No change in body weight. Reduced the TC, LDL-C.	[26]
Individuals with uncomplicated obesity; *n* = 46 (34 females, 12 males); Age = 42.9 ± 10 years; Average BMI = 34.2 ± 3.1 kg/m^2^	Open-label study	Anthocyanins and prebiotic powder mix	One sachet of powder containing 618 mg of black rice extract (120 mg of anthocyanins), 202 mg of black currant extract (60 mg of anthocyanins), 144 mg of European blueberry extract (35 mg of anthocyanins), and prebiotic fibers (1.9 g of inulin, 1.1 g of fructooligosaccharides) per day; 8 weeks.	Reduced firmicutes and actinobacteria load. Increased Bacteroidetes content. Improved bowel habits and intestinal ecosystem.	[28]
Overweight/obese individuals; Average BMI ≥ 25 kg/m^2^ Blueberry group, *n* = 27 (70% females, 30% males); Age = 34.9 ± 9.7 years; Control group, *n* = 27 (80% females, 20% males); Age = 32.2 ± 9.7 years	Double-blind, placebo-controlled trial	Blueberry	After 1st six weeks of nutrition therapy, 50 g of carbohydrate source was replaced with 50 g of blueberries in the daily diet; 12 weeks of nutrition therapy for both groups	Reduced body weight and body fat. Reduced TC, LDL-C significantly.	[29]
Mildly hypertensive overweight/obese individuals; QC group, *n* = 15 out of 16 (8 females, 8 males); Age = 47 ± 11 years; Average BMI = 31 ± 5 kg/m^2^; SBP = 142 ± 7 mmHg; DBP = 92 ± 4 mmHg; Placebo group, *n* = 14 out of 16 (8 females, 8 males); Age = 38 ± 14 years; Average BMI = 32 ± 5 kg/m^2^; SBP = 139 ± 5 mmHg; DBP = 91 ± 3 mmHg	Randomized, double-blind study	Queen Garnet (QC) plum juice; Placebo (raspberry cordial drink)	250 mL per day; QC juice contains C3G (78 mg C3GE/100 mL), C3R (24 mg C3GE/100 mL), quercetin glycosides (36 mg/100 mL); placebo drink contains C3G (0.1 mg C3GE/100 mL), C3R (0.1 mg C3GE/100 mL), quercetin glycosides (0.4 mg/100 mL);12 weeks	Reduced the LDL level. Reduced plasma glucose, insulin, and C-peptide, leptin and GLP-1 concentrations. Increased adiponectin content. Reduced the levels of IL-2, IL-6, IL-13, and TNF-α.	[30]
Obese individuals; Juҫara group, *n* = 13 (8 females, 5 males); Age = 45.76 ± 2.58 years; Average BMI = 34.63 ± 1.2 kg/m^2^; Placebo group, *n* = 14 (8 females, 6 males); Age = 45.07 ± 3.42 years; Average BMI = 33.82 ± 0.71 kg/m^2^	Randomized, double-blind controlled study	Juҫara berry (freeze-dried pulp); Placebo (maltodextrin)	5 g per day; 6 weeks	Reduced the TLR4, IL-6, MYD88, TNF-α, and MCP-1 level. Increased Ob-R protein and IL-10.	[32]
Lean individuals, *n* = 12 (6 females, 6 males); Age = 33 ± 3.2 years; Average BMI = 22.8 ± 0.6 kg/m^2^; Overweight individuals, *n* = 9 (4 females, 5 males); Age = 49.9 ± 4.2 years; Average BMI = 27.7 ± 0.4 kg/m^2^; Obese individuals, n = 8 (4 females, 4 males); Age = 43.3 ± 4.5 years; Average BMI = 34.3 ± 0.9 kg/m^2^	Open-label study	MEDOX^®^ capsules (Medapalett Pharmaceuticals, Biolink, Sandnes, Norway) containing purified anthocyanins of blackcurrant and bilberries	320 mg per 4 capsules per day; 2 capsules consumed twice daily along with usual diet; each capsule contains 3-O-rutinoside of cyanidin and delphinidin (1%), and 3-O-β-galactosides, 3-O-β-glucosides and 3-O-β-arabinosides of cyanidin (33%), delphinidin (58%), malvidin (3%), petunidin (2.5%) and peonidin (2.5%); 28 days	Reduced plasma CCL2, and IL-6 level	[33]

BMI: Body mass index; SBP: Systolic blood pressure; DBP: Diastolic blood pressure; C3G: Cyanidin 3-glucoside; C3GE: Cyanidin 3-glucoside equivalents; C3R: Cyanidin 3-rutinoside; TC: Total cholesterol; LDL-C: Low-density lipoproteins-cholesterol; IL: interleukin; TNF: Tumor necrosis factor; GLP-1: Glucagon-like peptide-1; MYD88: Myeloid differentiation primary response 88; TLR4: Toll-like receptor 4; MCP-1: Monocyte chemoattractant protein-1; Ob-R protein: Leptin receptor; CCL2: C-C Motif Chemokine Ligand 2.

**Table 2:** The beneficial effect of anthocyanins: Results of in vivo studies.

Model	Intervention	Dose	Duration	Results	Reference
High-fat-diet-induced obese mice	Grape pomace extract	250 mg/kg/day	12 weeks	Reduced the plasma C-reactive protein level.	[34]
High-fat-diet-fed hamsters	Mulberry water extracts	0.5 or 1.0 or 2.0% in diet	12 weeks	Reduced body weight and visceral fat. Reduced the TG, TC, LDL/HDL ratio, free fatty acids level, and hepatic lipids. Induced the hepatic carnitine palmitoyltransferase-1, and peroxisome proliferator-activated receptor α expressions. Reduced the HMG-CoA reductase, and fatty acid synthase expressions.	[35]
Male Zucker fatty rats	Freeze dried whole highbush blueberry powder	2.0% (wt/wt) in high fat (45% of kcal) diet	90 days	Reduced the TG, fasting insulin, HOMA-IR, glucose area under curve values, and abdominal fat mass. Increased PPAR activity and affected the PPAR expression associated with fat and glucose oxidation.	[36]
High-fat-diet-fed mice	Blueberry and Mulberry Juice	Average amount of blueberry juice (4.83 ± 0.36 mL per day per mouse) or mulberry juice (4.56 ± 0.57 mL per day per mouse) consumption	12 weeks	Inhibited body weight gain. Decreased serum cholesterol and leptin secretion. Reduced insulin resistance and lipid accumulation.	[37]
High-fat-diet-fed mice	Mulberry anthocyanins	40 or 200 mg/kg of food	12 weeks	Inhibited body weight gain. Reduced the resistance to insulin, leptin secretion, adipocytes size, and lipid accumulation.	[38]
KK-Ay mice	Cyanidin-3-*O*-*β*-D-glucoside (C3G)	1 g/kg of body weight	12 weeks	Reduced body weight, liver, and visceral adipose tissue weight. Reduced the plasma and hepatic TG, and steatosis score. Increased the pAMPK in skeletal muscle and visceral adipose. Stimulated the lipoprotein lipase activity.	[39]
Obese mice	Purple sweet potato-anthocyanins	200 mg/kg body weight/day	4 weeks	Reduced weight gain, TG, TC. Reduced the TBARSs content. Reduced the lipid accumulation. Increased the pAMPK and pACC activity.	[40]
Obese mice	C3G-rich purple corn color	2 g/kg in the diet	12 weeks	Reduced weight gain, and adipose tissue weight. Normalized the expression of TNF-α. Suppressed fatty acid and triacylglycerol synthesis.	[41]
High-fat-diet-fed mice	Purple corn extract	290 mg of anthocyanin concentration/kg/day	12 weeks	Reduced inflammatory mediators and attenuated adipose tissue inflammation.	[42]
Wistar rats	Anthocyanins-rich chokeberry extract	100 or 200 mg/kg body weight/day	6 weeks	Reduced epididymal fat, TC, LDL-C, blood glucose. Suppressed the expression of inflammatory cytokines, plasma TNF-α and IL-6. Increased expressions of *Gys1, Irs1, Irs2, Pi3k, Glut1, Glut4*, *Pparγ*, adiponectin, and zinc finger protein. Suppressed the expressions of *Fabp4, Fas,* and *Lpl.*	[43]
Sprague-Dawley Rat	Anthocyanins	6 or 24 mg/kg	40 days	Lowered body weight and food consumption. Reduced adipose tissue volume, and expression of neuropeptide Y. Increased expression of GABA-receptor Suppressed the expressions of PKA, and p-CREB.	[44]
Fat diet fed fischer rats	Anthocyanins grape-bilberry rich juice	1551 mg/L	10 weeks	Reduced serum cholesterol, TG, leptin, and resistin. Decreased saturated fatty acids and increased polyunsaturated fatty acids level.	[45]
High fat diet fed Rabbits	Blackcurrant pomace	1.5% in the diet	4 weeks	Reduced the putrefactive metabolites and β-glucuronidase activity. Reduced the TG, TC, LDL-C and free fatty acid content. Increased antioxidant activity.	[46]
High fat diet fed mice	Black soybean testa	1 g/kg bodyweight	12 weeks	Reduced food consumption, fat accumulation, lipogenesis, and inflammation. Increased lipolysis.	[47]
Rats	Aronia fruit-anthocyanins	0.4% in the diet	4 weeks	Reduced visceral fat mass, perirenal, mesenteric and epididymal adipose mass. Reduced fasting glucose level, TG, and suppressed the increase in blood glucose level.	[48]
High fat diet fed mice	Bilberry extract	0.1%	24 weeks	Reduced the expression of *Tnf* and regulated the neutrophil chemoattractant mKC.	[49]
High fat diet fed rat	Cranberry extract	200 mg/kg	30 days	Reduced body weight gain, TG, hepatic cholesterol content, fatty acid synthase, hypocorticosteronemia, lipid peroxidation, protein carbonylation, and fat accumulation.	[50]
High fat diet induced obese mice	Blueberry anthocyanins	50 or 100 or 200 mg/kg	8 weeks	Reduced body weight, serum glucose level, and expression of TNF-α, IL-6, PPARγ, and FAS genes. Decreased epididymal adipocytes and improved the serum and liver lipid profiles.	[51]
High fat diet fed mice	Mulberry and cherry anthocyanins	200 mg/kg	8 weeks	Reduced body weight, serum glucose and leptin levels, and expression of TNF-α, IL-6, iNOS, and NF-кB genes. Improved lipid profiles, and SOD and GPx activities. Reduced the production of MDA.	[52]
High fat diet fed rats	Blueberry	10% in feed	8 weeks	Increased *Gammaproteobacteria* abundance. Retained ileal villus height. Regularized the expression of TNF-α and IL-1β. Improved insulin signaling	[53]
High fat diet fed mice	whole berries	400 μg of anthocyanins equivalent/g	12 weeks	Improved body composition, energy expenditure, mitochondrial respiration. Reduced metabolic stress.	[54]
High fat diet fed mice	*Acanthopanax senticosus* fruits	0.5 or 1.0 g/kg	12 weeks	Improved glucose tolerance and insulin sensitivity. Reduced insulin level and lipid accumulation. Reduced the expression of FAS and induced the expression of cholesterol 7-α-hydroxylase. Increased pAMPK.	[55]
High fat diet fed mice	Sweet cherry anthocyanins	200 mg/kg	15 weeks	Nullified diet-induced hepatic steatosis	[56]
High fat diet fed mice	Mulberry ethanol extract	100 mg/kg	14 weeks	Reduced weight gain. Improved hepatic steatosis, adipose hypertrophy, and insulin resistance. Altered lipid and cholesterol metabolism.	[57]
Obese Zucker rats	Freeze dried baked purple potatoes	450 g/kg diet	8 weeks	Improved glucose tolerance and insulin sensitivity. Controlled blood pressure.	[58]
High fat diet fed mice	*Vitis* *vinifera* L. grape skin extract	200 mg/kg per day	12 weeks	Prohibited weight gain, insulin resistance, and dyslipidemia. Reduced oxidative stress	[59]
Obese diabetic Mice	Red raspberry fruit	5.3% freeze-dried raspberry	8 weeks	Improved antioxidant activity. Increased GPx activity	[60]
High fat diet fed mice	Purple sweet potato color	700 mg/kg/day	20 weeks	Prevented obesity and liver damages. Reduced oxidative stress. Suppressed NF-κB, NOD1/2 signaling, NLRP3 inflammasome activation. Restored NAD^+^ level.	[61]
Obese mice	C3G	1 mg/mL	16 weeks	Reduced weight gain, increased energy expenditure, retained glucose homeostasis, normalized hepatic steatosis and enhanced cold tolerance. Induced the formation of brown-like adipocytes. Influenced the expression of uncoupling protein 1 (UCP1).	[62]
High fructose and high fat diet induced obese mice	C3G	1 mg/mL	15 weeks	Improved energy expenditure and thermogenic capacity. Increased the expression of UCP1.	[63]
High fat diet fed mice	Blackberry or blueberry anthocyanins	200 mg/kg	12 weeks	Reduced lipid level, increased the SOD, GPx activity. Increased fecal SCFs level, suppressed the expression of IL-6, NF-κB, TNF-α. Affected the glycerophospholipid, glutathione metabolism, and the insulin-signaling pathway.	[64]
High fructose and high fat diet induced obese mice	Blueberry or C3G	6.4 g/kg/dayor 0.02 g/kg/day	8 weeks	Reduced body weight, body fat, and blood pressure. Improved glucose tolerance.	[65]
High fat diet fed mice	Anthocyanins mix	2 or 20 or 40 mg/kg	14 weeks	Diminished diet-induced obesity, dyslipidemia, insulin resistance, inflammation, and liver lipid deposition. Suppressed oxidative stress, NF-κB, and JNK activation, and PTP1B overexpression.	[66]
High fat diet fed rat	blackberry anthocyanin-rich extract	25 mg/kg	17 weeks	Improved the gut microbiota composition. Exhibited neuroprotective activity and improved tryptophan metabolism.	[67]
High sucrose and high fat diet induced obese mice	*Plinia jaboticaba* (Vell.) Berg extract	50 mg GAE/kg	8 weeks	Prohibited body weight gain and increase in FBG. Diminished hyperinsulinemia and high TC.	[68]
High fat diet induced obese mice	Anthocyanins	40 mg/kg	14 weeks	Improved intestinal permeability and endotoxemia. Normalized NADPH oxidase expression and NOS2 level, redox signaling, NF-κB, and ERK activation, and reduced oxidative stress. Reestablished microbial composition and restored MUC2 level.	[69]
High carbohydrate and high fat diet fed rat	*Davidsonia pruriens*	8 mg of anthocyanins equivalent/kg	8 weeks	Reduced fat mass, fat accumulation, retroperitoneal dipocytes level, TG, collagen deposition, and inflammation	[70]
High fat diet induced obese mice	*Aronia melanocarpa* extract	50 or 100 or 200 mg/kg	8 weeks	Suppressed weight gain and fat mass. Reduced TG, TC, LDL-C, leptin, insulin level. Suppressed adipogenesis.	[71]
High fat diet induced obese mice	Tart cherry extract	60 mg/kg in drinking water	6 weeks	Reduced IL-6 and leptin levels and improved the antioxidant capacity of the host.	[72]
High fat diet induced obese mice	*Aristotelia chilensis*	20 mg/mL in water	16 weeks	Suppressed weight gain. Improved the expression of genes associated with lipogenesis, fatty acid oxidation, thermogenesis.	[73]

TG: Triglyceride; TC: Total cholesterol; LDL: Low-density lipoproteins; HDL: High-density lipoproteins; HMG-CoA: 3-hydroxy-3-methylglutaryl-coenzyme A; HOMA-IR: Homeostasis model index of insulin resistance; PPAR: Peroxisome Proliferator-Activated Receptor; pAMPK: Adenosine monophosphate-activated protein kinase phosphorylation; TBARSs: Thiobarbituric acid reactive substances; pACC: Acetyl-coenzyme A carboxylase phosphorylation; TNF-α: Tumor necrosis factor-α; IL-6: interleukin-6; GABA: γ-aminobutyric acid; PKA: Protein kinase A-α; p-CREB: Phosphorylated cAMP-response element-binding protein; PPARγ: Peroxisome proliferator-activated receptor-γ; FAS: Fatty acid synthase; SOD: Superoxide dismutase; GPx: Glutathione peroxidase; MDA: Malondialdehyde; NF-κB: nuclear factor-κB; NAD^+^: Nicotinamide adenine dinucleotide; NOD1/2: Nucleotide oligomerization domain protein1/2; NLRP3: NOD-, LRR- and pyrin domain-containing protein 3; UCP1: Uncoupling protein 1; SCFs: Short-chain fatty acids; GAE: Gallic acid equivalent; FBG: Fasting blood glucose.

**Table 3:** The beneficial effect of anthocyanins: results of in vitro studies.

Model	Intervention	Dose	Results	Reference
Rat adipocytes	Cyanidin or Cyanidin 3-*O*-β-D-glucose (C3G) glucoside	100 µM	Increased the secretion of adiponectin and leptin, and expression of adipocyte-specific genes.	[74]
3T3-L1 adipocytes	Cyanidin or C3G	10–100 µM	Suppressed the MCP-1 and MRP-2 activation and release.	[75]
3T3-L1 adipocytes	C3G	20–100 µM	Suppressed the FFAs and glycerol release. Increased AMP-activated protein kinase activity. Decreased glutamine: fructose 6-phosphate aminotransferase activity. Decreased the adipose triglyceride lipase expression.	[76]
3T3-L1 adipocytes	Grape anthocyanins	1–40 µg/mL	Reduced the TG accumulation. Altered the lipogenic transcription factors.	[77]
3T3-L1 pre-adipocytes	Anthocyanins from *Vitis coignetiae* Pulliat	25–400 μg/mL	Attenuated the pre-adipocytes differentiation. Activated AMPK and suppressed the expression of adipogenic transcription factors.	[78]
3T3-L1 adipocytes	Delphinidin-3-*O*-β-glucoside	25, 50, and 100 µM	Reduced lipid accumulation. Suppressed adipogenic and lipogenic genes. Promoted lipid metabolism.	[79]
3T3-L1 pre-adipocytes	Purple corn silk extract	250–1000 μg/mL	Inhibits adipocyte proliferation. Reduced lipid accumulation	[80]
Hepatocytes (HB-8965^®^)	5% (v/v) of Raspberry polyphenolic extract	0.5 nmol/L	Modulated hepatic immune-metabolic mechanisms. Altered the endosome/lysosome activity.	[81]
HUVEC and 3T3-L1 cells	Anthocyanins from *Cornus kousa*	5–200 μg/mL	Suppressed angiogenesis and lipid accumulation.	[82]
3T3-L1 adipocytes	Anthocyanins	10 and 20 μg/mL	Reduced lipid accumulation and PPAR- γ level. Nullified the palmitic acid-induced NF-κB activation and adipocyte dysfunction related hypertrophy.	[83]
Fluorometric method	Cyanidin and C3G	-	Inhibits pancreatic lipase activity	[84]

MCP-1: Monocyte chemoattractant protein-1; MRP-2: Macrophage inflammatory protein-related protein-2; FFAs: Free fatty acids; TG: Triglyceride; AMPK: AMP-activated protein kinase; HUVEc: Human umbilical vein endothelial cell; PPAR-γ: Peroxisome proliferators-activated receptor-γ.

## References

[1] Ng M., Fleming T., Robinson M., Thomson B., Graetz N., Margono C., Mullany E.C., Biryukov S., Abbafati C., Abera S.F. (2014). Global, regional, and national prevalence of overweight and obesity in children and adults during 1980e2013: A systematic analysis for the Global Burden of Disease Study 2013. Lancet.

[2] Obesity and overweight. https://www.who.int/news-room/fact-sheets/detail/obesity-and-overweight.

[3] Sivamaruthi B.S., Kesika P., Suganthy N., Chaiyasut C. (2019). A review on role of microbiome in obesity and antiobesity properties of probiotic supplements. Biomed Res. Int..

[4] Mandviwala T., Khalid U., Deswal A. (2016). Obesity and cardiovascular disease: A risk factor or a risk marker?. Curr. Atheroscler. Rep..

[5] Genser L., Mariolo J.R.C., Castagneto-Gissey L., Panagiotopoulos S., Rubino F. (2016). Obesity, type 2 diabetes, and the metabolic syndrome: Pathophysiologic relationships and guidelines for surgical intervention. Surg. Clin. N. Am..

[6] Li L., Liu D.W., Yan H.Y., Wang Z.Y., Zhao S.H., Wang B. (2016). Obesity is an independent risk factor for non-alcoholic fatty liver disease: Evidence from a meta-analysis of 21 cohort studies. Obes. Rev..

[7] Harper J.W., Zisman T.L. (2016). Interaction of obesity and inflammatory bowel disease. World J. Gastroenterol..

[8] Donohoe C.L., Lysaght J., O’Sullivan J., Reynolds J.V. (2017). Emerging concepts linking obesity with the hallmarks of cancer. Trends Endocrinol. Metab..

[9] Bonfrate L., Wang D.Q., Garruti G., Portincasa P. (2014). Obesity and the risk and prognosis of gallstone disease and pancreatitis. Best Pract. Res. Clin. Gastroenterol..

[10] Martin-Jiménez C.A., Gaitán-Vaca D.M., Echeverria V., González J., Barreto G.E. (2017). Relationship between obesity, Alzheimer’s disease, and Parkinson’s disease: An astrocentric view. Mol. Neurobiol..

[11] Wirth A., Wabitsch M., Hauner H. (2014). The prevention and treatment of obesity. Dtsch. Arztebl. Int..

[12] Akhlaghi M., Ghobadi S., Mohammad Hosseini M., Gholami Z., Mohammadian F. (2018). Flavanols are potential anti-obesity agents, a systematic review and meta-analysis of controlled clinical trials. Nutr. Metab. Cardiovasc. Dis..

[13] Azzini E., Giacometti J., Russo G.L. (2017). Antiobesity effects of anthocyanins in preclinical and clinical studies. Oxid. Med. Cell. Longev..

[14] Kawser Hossain M., Abdal Dayem A., Han J., Yin Y., Kim K., Kumar Saha S., Yang G.M., Choi H.Y., Cho S.G. (2016). Molecular mechanisms of the anti-obesity and anti-diabetic properties of flavonoids. Int. J. Mol. Sci..

[15] Sivamaruthi B.S., Kesika P., Chaiyasut C. (2018). Anthocyanins in Thai rice varieties: Distribution and pharmacological significance. Int. Food Res. J..

[16] Najafabadi N.S., Sahari M.A., Barzegar M., Esfahani Z.H. (2017). Effects of concentration method and storage time on some bioactive compounds and color of jujube (*Ziziphus jujuba* var *vulgaris*) concentrate. J. Food Sci. Technol..

[17] Alighourchi H., Barzegar M., Abbasi S. (2008). Anthocyanins characterization of 15 Iranian pomegranate (*Punica granatum* L.) varieties and their variation after cold storage and pasteurization. Eur. Food Res. Technol..

[18] Hasnaoui N., Jbir R., Mars M., Trifi M., Kamal-Eldin A., Melgarejo P., Hernandez F. (2011). Organic acids, sugars, and anthocyanins contents in juices of Tunisian pomegranate fruits. Int. J. Food Prop..

[19] Batista A.G., da Silva J.K., Cazarin C.B.B., Biasoto A.C.T., Sawaya A.C.H.F., Prado M.A., Júnior M.R.M. (2017). Red-jambo (*Syzygium malaccense*): Bioactive compounds in fruits and leaves. LWT Food Sci. Technol..

[20] Khoo H.E., Azlan A., Tang S.T., Lim S.M. (2017). Anthocyanidins and anthocyanins: Colored pigments as food, pharmaceutical ingredients, and the potential health benefits. Food Nutr. Res..

[21] Zhang J., Celli G.B., Brooks M.S., Brooks M.S.-L., Celli G.B. (2019). Natural sources of anthocyanins. Anthocyanins from Natural Sources: Exploiting Targeted Delivery for Improved Health.

[22] Sivamaruthi B.S., Kesika P., Subasankari K., Chaiyasut C. (2018). Beneficial effects of anthocyanins against diabetes mellitus associated consequences-A mini review. Asian Pac. J. Trop. Biomed..

[23] Bertoia M.L., Rimm E.B., Mukamal K.J., Hu F.B., Willett W.C., Cassidy A. (2016). Dietary flavonoid intake and weight maintenance: Three prospective cohorts of 124 086 US men and women followed for up to 24 years. BMJ.

[24] Dugo P., Mondello L., Morabito D., Dugo G. (2003). Characterization of the anthocyanin fraction of sicilian blood orange juice by micro-HPLC-ESI/MS. J. Agric. Food Chem..

[25] Silveira J.Q., Dourado G.K.Z.S., Cesar T.B. (2015). Red-fleshed sweet orange juice improves the risk factors for metabolic syndrome. Int. J. Food Sci. Nutr..

[26] Azzini E., Venneria E., Ciarapica D., Foddai M.S., Intorre F., Zaccaria M., Maiani F., Palomba L., Barnaba L., Tubili C. (2017). Effect of red orange juice consumption on body composition and nutritional status in overweight/obese female: A pilot study. Oxid. Med. Cell. Longev..

[27] Scordino M., Sabatino L., Lazzaro F., Borzì M.A., Gargano M., Traulo P., Gagliano G. (2015). Blood orange anthocyanins in fruit beverages: How the commercial shelf life reflects the quality parameter. Beverages.

[28] Hester S.N., Mastaloudis A., Gray R., Antony J.M., Evans M., Wood S.M. (2018). Efficacy of an anthocyanin and prebiotic blend on intestinal environment in obese male and female subjects. J. Nutr. Metab..

[29] Istek N., Gurbuz O. (2017). Investigation of the impact of blueberries on metabolic factors influencing health. J. Funct. Foods.

[30] Bhaswant M., Brown L., Mathai M.L. (2019). Queen Garnet plum juice and raspberry cordial in mildly hypertensive obese or overweight subjects: A randomized, double-blind study. J. Funct. Foods.

[31] Bicudo M.O.P., Ribani R.H., Beta T. (2014). Anthocyanins, phenolic acids and antioxidant properties of juçara fruits (*Euterpe edulis* M.) along the on-tree ripening process. Plant Foods Hum. Nutr..

[32] Santamarina A.B., Jamar G., Mennitti L.V., Cesar H.C., Vasconcelos J.R., Oyama L.M., de Rosso V.V., Pisani L.P. (2019). Obesity-related inflammatory modulation by juçara berry (*Euterpe edulis* Mart.) supplementation in Brazilian adults: A double-blind randomized controlled trial. Eur. J. Nutr..

[33] Vugic L., Colson N., Nikbakht E., Gaiz A., Olivia J. (2020). Holland, Avinash Reddy Kundur, Indu Singh. Anthocyanin supplementation inhibits secretion of pro-inflammatory cytokines in overweight and obese individuals. J. Funct. Foods.

[34] Hogan S., Canning C., Sun S., Sun X., Zhou K. (2010). Effects of grape pomace antioxidant extract on oxidative stress and inflammation in diet induced obese mice. J. Agric. Food Chem..

[35] Peng C.H., Liu L.K., Chuang C.M., Chyau C.C., Huang C.N., Wang C.J. (2011). Mulberry water extracts possess an anti-obesity effect and ability to inhibit hepatic lipogenesis and promote lipolysis. J. Agric. Food Chem..

[36] Seymour E.M., Tanone I.I., Urcuyo-Llanes D.E., Lewis S.K., Kirakosyan A., Kondoleon M.G., Kaufman P.B., Bolling S.F. (2011). Blueberry intake alters skeletal muscle and adipose tissue peroxisome proliferator-activated receptor activity and reduces insulin resistance in obese rats. J. Med. Food.

[37] Wu T., Tang Q., Gao Z., Yu Z., Song H., Zheng X., Chen W. (2013). Blueberry and mulberry juice prevent obesity development in C57BL/6 mice. PLoS ONE.

[38] Wu T., Qi X., Liu Y., Guo J., Zhu R., Chen W., Zheng X., Yu T. (2013). Dietary supplementation with purified mulberry (*Morus australis* Poir) anthocyanins suppresses body weight gain in high-fat diet fed C57BL/6 mice. Food Chem..

[39] Wei X., Wang D., Yang Y., Xia M., Li D., Li G., Zhu Y., Xiao Y., Ling W. (2011). Cyanidin-3-O-β-glucoside improves obesity and triglyceride metabolism in KK-Ay mice by regulating lipoprotein lipase activity. J. Sci. Food Agric..

[40] Hwang Y.P., Choi J.H., Han E.H., Kim H.G., Wee J.H., Jung K.O., Jung K.H., Kwon K.I., Jeong T.C., Chung Y.C. (2011). Purple sweet potato anthocyanins attenuate hepatic lipid accumulation through activating adenosine monophosphate-activated protein kinase in human HepG2 cells and obese mice. Nutr. Res..

[41] Tsuda T., Horio F., Uchida K., Aoki H., Osawa T. (2003). Dietary cyanidin 3-O-beta-D-glucoside-rich purple corn color prevents obesity and ameliorates hyperglycemia in mice. J. Nutr..

[42] Tomay F., Marinelli A., Leoni V., Caccia C., Matros A., Mock H.P., Tonelli C., Petroni K. (2019). Purple corn extract induces long-lasting reprogramming and M2 phenotypic switch of adipose tissue macrophages in obese mice. J. Transl. Med..

[43] Qin B., Anderson R.A. (2012). An extract of chokeberry attenuates weight gain and modulates insulin, adipogenic and inflammatory signalling pathways in epididymal adipose tissue of rats fed a fructose-rich diet. Br. J. Nutr..

[44] Badshah H., Ullah I., Kim S.E., Kim T.H., Lee H.Y., Kim M.O. (2013). Anthocyanins attenuate body weight gain via modulating neuropeptide Y and GABAB1 receptor in rat’s hypothalamus. Neuropeptides.

[45] Graf D., Seifert S., Jaudszus A., Bub A., Watzl B. (2013). Anthocyanin-rich juice lowers serum cholesterol, leptin, and resistin and improves plasma fatty acid composition in Fischer rats. PLoS ONE.

[46] Jurgoński A., Juśkiewicz J., Zduńczyk Z., Matusevicius P., Kołodziejczyk K. (2014). Polyphenol-rich extract from blackcurrant pomace attenuates the intestinal tract and serum lipid changes induced by a high-fat diet in rabbits. Eur. J. Nutr..

[47] Kim S.Y., Wi H.R., Choi S., Ha T.J., Lee B.W., Lee M. (2015). Inhibitory effect of anthocyanin-rich black soybean testa (*Glycine max* (L.) Merr.) on the inflammation-induced adipogenesis in a DIO mouse model. J. Funct. Foods.

[48] Takahashi A., Shimizu H., Okazaki Y., Sakaguchi H., Taira T., Suzuki T., Chiji H. (2015). Anthocyanin-rich phytochemicals from aronia fruits inhibit visceral fat accumulation and hyperglycemia in high-fat diet-induced dietary obese rats. J. Oleo Sci..

[49] van der Heijden R.A., Morrison M.C., Sheedfar F., Mulder P., Schreurs M., Hommelberg P.P., Hofker M.H., Schalkwijk C., Kleemann R., Tietge U.J. (2016). Effects of anthocyanin and flavanol compounds on lipid metabolism and adipose tissue associated systemic inflammation in diet-induced obesity. Mediat. Inflamm..

[50] Peixoto T.C., Moura E.G., de Oliveira E., Soares P.N., Guarda D.S., Bernardino D.N., Ai X.X., Rodrigues V.D.S.T., de Souza G.R., da Silva A.J.R. (2018). Cranberry (*Vaccinium macrocarpon*) extract treatment improves triglyceridemia, liver cholesterol, liver steatosis, oxidative damage and corticosteronemia in rats rendered obese by high fat diet. Eur. J. Nutr..

[51] Wu T., Jiang Z., Yin J., Long H., Zheng X. (2016). Anti-obesity effects of artificial planting blueberry (*Vaccinium ashei*) anthocyanin in high fat diet-treated mice. Int. J. Food Sci. Nutr..

[52] Wu T., Yin J., Zhang G., Long H., Zheng X. (2016). Mulberry and cherry anthocyanin consumption prevents oxidative stress and inflammation in diet-induced obese mice. Mol. Nutr. Food Res..

[53] Lee S., Keirsey K.I., Kirkland R., Grunewald Z.I., Fischer J.G., de La Serre C.B. (2018). Blueberry supplementation influences the gut microbiota, inflammation, and insulin resistance in high-fat-diet-fed rats. J. Nutr..

[54] Skates E., Overall J., DeZego K., Wilson M., Esposito D., Lila M.A., Komarnytsky S. (2018). Berries containing anthocyanins with enhanced methylation profiles are more effective at ameliorating high fat diet-induced metabolic damage. Food Chem. Toxicol..

[55] Saito T., Nishida M., Saito M., Tanabe A., Eitsuka T., Yuan S.H., Ikekawa N., Nishida H. (2016). The fruit of *Acanthopanax senticosus* (Rupr. et Maxim.) Harms improves insulin resistance and hepatic lipid accumulation by modulation of liver adenosine monophosphate-activated protein kinase activity and lipogenic gene expression in high-fat diet-fed obese mice. Nutr. Res..

[56] Song H., Wu T., Xu D., Chu Q., Lin D., Zheng X. (2016). Dietary sweet cherry anthocyanins attenuates diet-induced hepatic steatosis by improving hepatic lipid metabolism in mice. Nutrition.

[57] Song H., Lai J., Tang Q., Zheng X. (2016). Mulberry ethanol extract attenuates hepatic steatosis and insulin resistance in high-fat diet-fed mice. Nutr. Res..

[58] Ayoub H.M., McDonald M.R., Sullivan J.A., Tsao R., Platt M., Simpson J., Meckling K.A. (2017). The effect of anthocyanin-rich purple vegetable diets on metabolic syndrome in obese Zucker rats. J. Med. Food.

[59] da Costa G.F., Santos I.B., de Bem G.F., Cordeiro V.S.C., da Costa C.A., de Carvalho L.C.R.M., Ognibene D.T., Resende A.C., de Moura R.S. (2017). The beneficial effect of anthocyanidin-rich *Vitis vinifera* L. grape skin extract on metabolic changes induced by high-fat diet in mice involves antiinflammatory and antioxidant actions. Phytother. Res..

[60] Noratto G.D., Chew B.P., Atienza L.M. (2017). Red raspberry (*Rubus idaeus* L.) intake decreases oxidative stress in obese diabetic (db/db) mice. Food Chem..

[61] Wang X., Zhang Z.F., Zheng G.H., Wang A.M., Sun C.H., Qin S.P., Zhuang J., Lu J., Ma D.F., Zheng Y.L. (2017). The inhibitory effects of purple sweet potato color on hepatic inflammation is associated with restoration of NAD^+^ levels and attenuation of NLRP3 inflammasome activation in high-fat-diet-treated mice. Molecules.

[62] You Y., Yuan X., Liu X., Liang C., Meng M., Huang Y., Han X., Guo J., Guo Y., Ren C. (2017). Cyanidin-3-glucoside increases whole body energy metabolism by upregulating brown adipose tissue mitochondrial function. Mol. Nutr. Food Res..

[63] You Y., Han X., Guo J., Guo Y., Yin M., Liu G., Huang W., Zhan J. (2018). Cyanidin-3-glucoside attenuates high-fat and high-fructose diet-induced obesity by promoting the thermogenic capacity of brown adipose tissue. J. Funct. Foods.

[64] Wu T., Gao Y., Guo X., Zhang M., Gong L. (2018). Blackberry and blueberry anthocyanin supplementation counteract high-fat-diet-induced obesity by alleviating oxidative stress and inflammation and accelerating energy expenditure. Oxid. Med. Cell. Longev..

[65] Shi M., Mathai M.L., Xu G., McAinch A.J., Su X.Q. (2019). The effects of supplementation with blueberry, cyanidin-3-O-β-glucoside, yoghurt and its peptides on obesity and related comorbidities in a dietinduced obese mouse model. J. Funct. Foods.

[66] Daveri E., Cremonini E., Mastaloudis A., Hester S.N., Wood S.M., Waterhouse A.L., Anderson M., Fraga C.G., Oteiza P.I. (2018). Cyanidin and delphinidin modulate inflammation and altered redox signaling improving insulin resistance in high fat-fed mice. Redox Biol..

[67] Marques C., Fernandes I., Meireles M., Faria A., Spencer J.P.E., Mateus N., Calhau C. (2018). Gut microbiota modulation accounts for the neuroprotective properties of anthocyanins. Sci. Rep..

[68] Moura M.H.C., Cunha M.G., Alezandro M.R., Genovese M.I. (2018). Phenolic-rich jaboticaba (*Plinia jaboticaba* (Vell.) Berg) extracts prevent high-fat-sucrose diet-induced obesity in C57BL/6 mice. Food Res. Int..

[69] Cremonini E., Daveri E., Mastaloudis A., Adamo A.M., Mills D., Kalanetra K., Hester S.N., Wood S.M., Fraga C.G., Oteiza P.I. (2019). Anthocyanins protect the gastrointestinal tract from high fat diet-induced alterations in redox signaling, barrier integrity and dysbiosis. Redox Biol..

[70] John O.D., Mouatt P., Prasadam I., Xiao Y., Panchal S.K., Brown L. (2019). The edible native Australian fruit, Davidson’s plum (*Davidsonia pruriens*), reduces symptoms in rats with diet-induced metabolic syndrome. J. Funct. Foods.

[71] Lim S.M., Lee H.S., Jung J.I., Kim S.M., Kim N.Y., Seo T.S., Bae J.S., Kim E.J. (2019). Cyanidin-3-O-galactoside-enriched Aronia melanocarpa extract attenuates weight gain and adipogenic pathways in high-fat diet-induced obese C57BL/6 mice. Nutrients.

[72] Nemes A., Homoki J.R., Kiss R., Hegedűs C., Kovács D., Peitl B., Gál F., Stündl L., Szilvássy Z., Remenyik J. (2019). Effect of anthocyanin-rich tart cherry extract on inflammatory mediators and adipokines involved in type 2 diabetes in a high fat diet induced obesity mouse model. Nutrients.

[73] Sandoval V., Femenias A., Martínez-Garza Ú., Sanz-Lamora H., Castagnini J.M., Quifer-Rada P., Lamuela-Raventós R.M., Marrero P.F., Haro D., Relat J. (2019). Lyophilized Maqui (*Aristotelia chilensis*) berry induces browning in the subcutaneous white adipose tissue and ameliorates the insulin resistance in high fat diet-induced obese mice. Antioxidants.

[74] Tsuda T., Ueno Y., Aoki H., Koda T., Horio F., Takahashi N., Kawada T., Osawa T. (2004). Anthocyanin enhances adipocytokine secretion and adipocyte-specific gene expression in isolated rat adipocytes. Biochem. Biophys. Res. Commun..

[75] Choe M.R., Kang J.H., Yoo H., Choe S.Y., Yang C.H., Kim M.O., Yu R. (2007). Cyanidin and cyanidin-3-O-β-D-glucoside suppress the inflammatory responses of obese adipose tissue by inhibiting the release of chemokines MCP-1 and MRP-2. J. Food Sci. Nutr..

[76] Guo H., Guo J., Jiang X., Li Z., Ling W. (2012). Cyanidin-3-O-β-glucoside, a typical anthocyanin, exhibits antilipolytic effects in 3T3-L1 adipocytes during hyperglycemia: Involvement of FoxO1-mediated transcription of adipose triglyceride lipase. Food Chem. Toxicol..

[77] Lee B., Lee M., Lefevre M., Kim H.R. (2014). Anthocyanins inhibit lipogenesis during adipocyte differentiation of 3T3-L1 preadipocytes. Plant Foods Hum. Nutr..

[78] Han M.H., Kim H.J., Jeong J.W., Park C., Kim B.W., Choi Y.H. (2018). Inhibition of adipocyte differentiation by anthocyanins isolated from the fruit of *Vitis coignetiae* Pulliat is associated with the activation of AMPK signaling pathway. Toxicol. Res..

[79] Park M., Sharma A., Lee H.J. (2019). Anti-adipogenic effects of delphinidin-3-O-β-glucoside in 3T3-L1 preadipocytes and primary white adipocytes. Molecules.

[80] Chaiittianan R., Sutthanut K., Rattanathongkom A. (2017). Purple corn silk: A potential anti-obesity agent with inhibition on adipogenesis and induction on lipolysis and apoptosis in adipocytes. J. Ethnopharmacol..

[81] Fotschki B., Laparra J.M., Sójka M. (2018). Raspberry polyphenolic extract regulates obesogenic signals in hepatocytes. Molecules.

[82] Khan M.I., Shin J.H., Shin T.S., Kim M.Y., Cho N.J., Kim J.D. (2018). Anthocyanins from Cornus kousa ethanolic extract attenuate obesity in association with anti-angiogenic activities in 3T3-L1 cells by down-regulating adipogeneses and lipogenesis. PLoS ONE.

[83] Muscarà C., Molonia M.S., Speciale A., Bashllari R., Cimino F., Occhiuto C., Saija A., Cristani M. (2019). Anthocyanins ameliorate palmitate-induced inflammation and insulin resistance in 3T3-L1 adipocytes. Phytother. Res..

[84] Vijayaraj P., Nakagawa H., Yamaki K. (2019). Cyanidin and cyanidin-3-glucoside derived from *Vigna unguiculata* act as noncompetitive inhibitors of pancreatic lipase. J. Food Biochem..

